# Evaluation of the Efficacy of Paracetamol in the Control of Pain After Adenotonsillectomy in the Pediatric Population

**DOI:** 10.7759/cureus.30807

**Published:** 2022-10-28

**Authors:** Ricardo Costa, Ângelo Fernandes, Rui Fonseca

**Affiliations:** 1 Otolaryngology - Head and Neck Surgery, Hospital da Senhora da Oliveira, Guimarães, PRT

**Keywords:** monotherapy, paracetamol, postoperative pain, pain control, adenotonsillectomy

## Abstract

Introduction

Adenotonsillectomy is a common surgical procedure in otolaryngology. Over the years, several techniques have been developed and modified in order to reduce mortality and morbidity. Postoperative pain control remains controversial. The aim of this study was to evaluate the efficacy of paracetamol alone in the control of postoperative pain.

Methods

A prospective study was conducted between May 2018 and February 2019, including 76 pediatric patients (age < 18 years), who underwent adenotonsillectomy. The surgeries were performed by the lead author with the same surgical technique. Patients were evaluated one week and one month after surgery through the application of the visual analog pain scale and the number of days of pain was assessed by the need for medication.

Results

Seventy-six total adenotonsillectomy were performed, with a total of 152 tonsils removed. The majority of patients were male (n=39, 51.3%), with an average age of 6.9 years (min 5, max 15 years). The most frequent surgical indication was sleep breathing disorders, present in 86.9% of the cases. The average duration of postoperative pain was 3 days, with no significant difference between groups (p>0.05). The average intensity of postoperative pain was 3.36 and was higher in patients with infectious criteria as surgical indications (p<0.05). Postoperative bleeding occurred in 3.9% (n=3) of the children, self-limited, without the need for readmission or surgical revision.

Conclusion

Pain after adenotonsillectomy was more intense in patients undergoing surgery for infectious criteria. Paracetamol used in monotherapy has shown safety and efficacy in controlling postoperative pain.

## Introduction

Adenotonsillectomy is one of the most performed surgical procedures in children worldwide. Most cases are performed in an outpatient setting and, therefore, the management of postoperative pain usually occurs at home with medication given orally [1]. Adenotonsillectomy is mainly performed for the treatment of obstructive sleep-disordered breathing (oSDB) or recurrent tonsillitis. Currently, oSDB represents the most common surgical indication (almost 70% of cases) [2].

The American Academy of Otolaryngology - Head and Neck Surgery (AAO-HNS) defines tonsillectomy as a surgical procedure by which the palatine tonsil, including its capsule, is completely removed by dissecting the peritonsillar space between the tonsil capsule and the muscular wall. It can be performed alone or associated with other procedures, such as adenoidectomy [3].

There are several factors that influence the success of post-tonsillectomy pain control, from individual factors related to parents/caregivers, surgical technique and medication used [1]. Inadequate analgesia can cause dehydration, nausea, respiratory problems and hemorrhage, which can cause parents/caregivers anxiety and a return to the hospital service [4]. Several therapeutic strategies can be adopted to control postoperative pain and the most frequently used medications are paracetamol, ibuprofen and opioids.

Paracetamol is often used to control pain after tonsillectomy in children. However, there is concern about its effectiveness in optimizing pain control. In fact, studies show that pain may not be completely controlled, even with therapeutic combinations [1]. When comparing the use of paracetamol alone with paracetamol associated with codeine, there was no significant difference in the control of post-tonsillectomy pain [1,5].

The main advantage of paracetamol is its safety; however, there is potential for liver damage, especially if associated with high doses [1,6]. Intravenous paracetamol can be used safely and effectively in the immediate postoperative period, in order to avoid the use of opioids [1,7]. Non-steroidal anti-inflammatory drugs (NSAIDs), mainly ibuprofen, are often used to control pain. Ibuprofen has been shown to be effective in controlling post-tonsillectomy pain.

However, many surgeons remain concerned about post-tonsillectomy bleeding, despite systematic reviews showing no statistically significant difference in bleeding rates between NSAIDs, opioids and placebo, including bleeding requiring hospitalization or surgery [1,8,9]. Although the systematic reviews mentioned above have not shown a difference in bleeding rates with the use of NSAIDs, the severity of bleeding, as assessed by the volume of blood loss, need for transfusion or difficulty in hemostasis, remains under study [10]. The AAO-HNS recommends avoiding the use of ketorolac in the pediatric population due to post-tonsillectomy bleeding rates ranging from 4.4% to 18.0%. However, this increased bleeding risk appears to occur primarily in adults [11]. There is a great debate about the effect of opioids on postoperative respiratory depression, especially in the pediatric population with oSDB. The risk of respiratory depression associated with opioids is related to a change in a cytochrome P450 enzyme (CYP450) that leads to the rapid metabolism of codeine to morphine, causing respiratory depression [1]. It was also found that a subgroup of patients cannot metabolize codeine, which reduces its analgesic effect [1]. Children with oSDB are more sensitive not only to the respiratory side effect of opioids, but also to their analgesic effect, so they need a lower dose of opioids to obtain the same degree of analgesia [2,12]. The additional benefit of opioids for pain relief that was not controlled by acetaminophen and/or ibuprofen is not well documented, so other therapeutic approaches should be used to minimize the need for opioids [13].

Analgesic protocols based on the use of acetaminophen and NSAIDs as an alternative to opioids have been adopted in younger children and children with oSDB in several institutions, with studies demonstrating the achievement of satisfactory pain control with both acetaminophen and ibuprofen in monotherapy [1,14].

There are several surgical techniques and instruments for performing tonsillectomy, including cold and hot dissection, for total (extracapsular) or partial (intracapsular) removal of the tonsils and, at the instrument level, cold dissection tools, monopolar and bipolar electric scalpel, coblation devices, harmonic scalpel and argon plasma coagulation, among others [1].

Cold dissection is associated with a less painful postoperative period compared to hot dissection techniques. However, its use has been decreasing due to longer operative time and hemorrhagic risk [1,15]. In partial tonsillectomy (intracapsular), the pharyngeal constrictor muscles and the tonsil pillars are not affected, so the postoperative period is less painful and, consequently, there is less use of analgesia and a faster return to the normal diet [1,16].

Regarding adjuvant treatments, pre-incisional peritonsillar infiltration with local anesthetic and/or corticosteroid has been shown to provide complementary analgesia [17]. However, one review concluded that there was no scientific evidence to recommend the use of perioperative local anesthetics [18]. Intraoperative use of the cold saline solution is associated with pain reduction [19]. The aim of this study was to assess the safety and efficacy profile of paracetamol in monotherapy for the control of postoperative pain after tonsillectomy.

## Materials and methods

A prospective study was conducted in Hospital da Senhora da Oliveira - Guimarães, for a period of 10 months, between May 2018 and February 2019, including 76 pediatric patients (age < 18 years), who underwent extracapsular tonsillectomy associated with adenoidectomy with or without myringotomy with placement of a transtympanic ventilation tube, to assess the safety and efficacy profile of paracetamol in monotherapy for the control of postoperative throat pain.

All patients were submitted to adenotonsillectomy with cold dissection, extracapsular, and under general anesthesia with orotracheal intubation. All the surgeries were performed by the lead author. Every patient in this study met the criteria for surgical intervention based on the 2019 AAO-HNS guidelines, such as obstructive sleep-disordered breathing or acute recurrent infections [3].

During surgery, anesthesia was administered by inhalation of O_2_/air and sevoflurane (1.5-3 vol%), intravenous propofol (2-3 mg/kg), fentanyl (2-3 micrograms/kg), dexamethasone (0 .2 mg/kg), paracetamol 15 to 20 mg/kg, tramadol 1-2 mg/kg, ondansetron 0.1 mg/kg and sugammadex 2 mg/kg. Patients started a cold liquid diet six hours after surgery. In the outpatient regimen, all patients remained under observation for a period of up to 24 hours, with clinical discharge without any recorded complications.

After surgery, each patient/parent/caregiver was given the same recommendations, to administer analgesia at a fixed time, with paracetamol at a dose of 10 to 15 mg/kg/dose every 6 hours (maximum 100 mg/kg/day or 4 g/day if >50 kg). All the orientations were given by the doctor and reinforced by a nurse, in addition to the written instructions given to each family. The recommended diet was a soft and cold diet until the evaluation at the first appointment.

Patients were evaluated seven and 30 days after surgery. At the first appointment after surgery, patients/parents/caregivers were asked about throat pain intensity and duration. Children were assessed for their pain intensity, with the help of their parents/caregivers, using the visual analog scale (VAS). 

Information and explanations on how VAS works were provided to the child at the time of the assessment. The VAS was presented as a 10 cm horizontal line with two ends (0: no pain and 10: worst possible pain) to both parents/caregivers and the child. The children, with the help of their parents/caregivers, were asked by the author to place a mark at the point where they thought it represented the intensity of their pain. Pain duration was evaluated by parents/caregivers as the number of days in which analgesic medication was required. Complications, such as nausea, vomiting, bleeding or fever, were recorded during the follow-up period.

This study was conducted in a manner that warrants the confidentiality of all included patients. Data collected had been deidentified prior to being stored. Permission was granted by the institution’s ethical committee before starting the study and the data collection. Written informed consent was obtained from the parents of the patients for participation in this study.

Statistical analysis was performed using the Statistical Package for the Social Sciences® (SPSS V.25.0) program, with the application of a t-test for independent variables. The level of statistical significance adopted was p < 0.05.

## Results

Seventy-six patients were included in the study, 39 (51.3%) male and 37 (48.7%) female, with an average age of 6.9 years (min 5 years; max 15 years). Patients without pathological history and usual medication were included in the study. All of the patients did not have any history of respiratory infections or use of antibiotics for at least two weeks before surgery.

**Figure 1:**
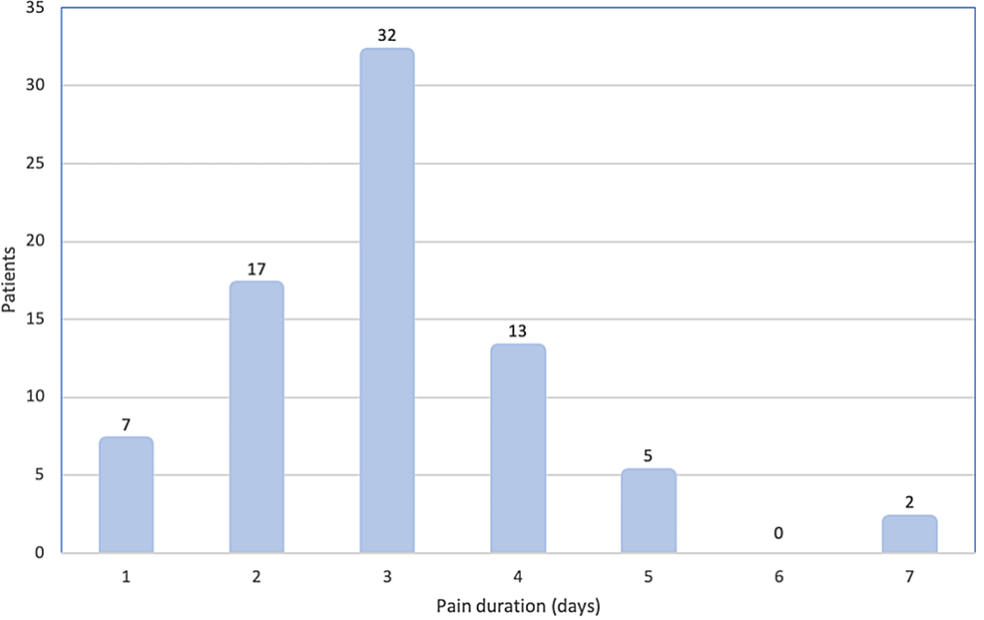
Pain duration evaluated as the number of days in which analgesic medication was required

The most frequent surgical indication was OSAS, present in 86.9% (n=66) of the cases. The history of recurrent tonsillitis as an isolated surgical criterion was present in 13.1% of the cases (n=10) and in 40.8% of the cases (n=31) when associated with OSAS. The average duration of postoperative pain was three days and 81.6% of patients had pain for two to four days (Figure 1).

**Figure 2:**
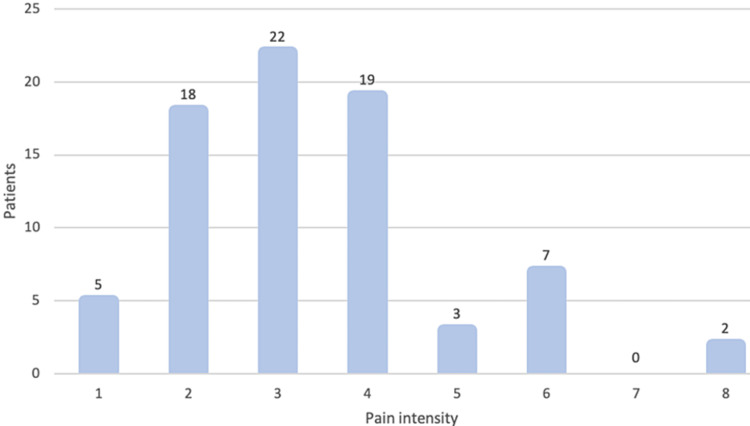
Pain intensity assessed using the VAS

There was no statistically significant difference in the duration of postoperative pain between the gender and surgical indication groups (p>0.05). The average pain intensity was 3.4, with 77.6% (n=59) of patients presenting pain intensity between two and four (Figure 2).

Pain intensity was higher in patients with infectious criteria (p<0.05) and there was no statistically significant difference between the gender group (p>0.05). Regarding the presence of complications, bleeding was recorded in 3.9% of the children (n=3), which was self-limited, without the need for new hospitalization or surgical revision.

## Discussion

Adenotonsillectomy is the second most performed surgical procedure in the pediatric age. Postoperative recovery is strongly associated with pain intensity and underlying functional limitations [20]. Considering that it is preferably performed on an outpatient regimen, pain management in the postoperative period is one of the main concerns, with an impact on feeding, recovery time and return to daily activities.

The VAS presents itself as a reliable measure for pain assessment (self-assessment) and has been widely used. VAS values ​​were sensitive to changes in pain levels during the postoperative period and after analgesia and showed a significant correlation with parents' ratings of children's pain [14,21].

In this study, VAS was used to assess the efficacy of paracetamol in the treatment of pain after extracapsular tonsillectomy by cold dissection. Other medications, such as NSAIDs or opioids, were not used in this sample. Pain intensity can also be indirectly assessed by other measures, such as returning to a normal diet, refusing fluids, nausea, or the use of antibiotics [14].

Paracetamol has a central analgesic effect that results from the activation of descending serotoninergic pathways, by inhibition of prostaglandin synthesis or indirect activation of cannabinoid receptors. Paracetamol has a weak anti-inflammatory effect, little or no gastrointestinal side effects, and causes only a small dose-dependent change in platelet function [14].

Ibuprofen has become the main NSAID used for postoperative analgesia, achieving adequate pain control. However, their use remains controversial, since NSAIDs can also cause platelet dysfunction, which can lead to an increased risk of postoperative bleeding [14].

The administration scheme of analgesics remains a controversial topic in the literature. Fixed regimen medication is widely used, as adopted in this study. A fixed administration plan is especially necessary to obtain the optimal analgesic effect of paracetamol in monotherapy [14].

In our sample, the average duration of postoperative pain was 3 days, with 81.6% of the cases having pain for two to four days, with an average intensity of 3.4, which demonstrates that paracetamol can be administered safely and relatively effectively as monotherapy.

In our study, no statistically significant association was observed between gender, intensity and duration of postoperative pain, unlike other studies in the literature [22,23]. However, there was a statistically significant association between the intensity of postoperative pain and the presence of recurrent tonsillitis.

Postoperative tonsillectomy pain has its own natural course. There are studies that have verified that in children, regardless of the analgesic and dosage regimen used, pain is classified as moderate to severe, with VAS values ​​greater than 5 in the first seven days [14].

After seven days, the rating of pain intensity decreases significantly [14,24]. Regarding complications, all cases of bleeding occurred in male patients who had surgery because of recurrent tonsillitis, described in the literature as a risk factor for hemorrhage, however, without statistical significance in our study.

## Conclusions

There is still no consensus on the most effective postoperative pain control plan after tonsillectomy. In addition, pain assessment methods in studies are very heterogeneous, especially for the pediatric population. However, our study demonstrated that adequate pain control can be achieved with paracetamol alone, without undesired complications.
